# Supplementary material for: Is It Just Face Blindness? Exploring Developmental Comorbidity in Individuals with Self-Reported Developmental Prosopagnosia
Source: Brain Sci. 2022 Feb 8;12(2):230. doi: 10.3390/brainsci12020230 (PMC8870183; doi:10.3390/brainsci12020230)
Supplement: Supplementary file 1 [file brainsci-12-00230-s001.zip › brainsci-1536071-supplementary.pdf]

The Difficulties and Abilities in Developmental Prosopagnosia Questionnaire (DAP-Q)

This appendix holds an overview of the items from the DAP-Q alongside the introductory information about the project for potential participants to consider as well as the last pages of the questionnaire for full and part completion.

Participant Project Information (First Page of the Questionnaire)

Difficulties and Abilities in Developmental Prosopagnosia

This project aims to investigate developmental prosopagnosia, and difficulties and abilities which might be related to this condition. When one has developmental prosopagnosia, one has severe difficulties recognising faces, which might make several aspects of social life challenging.
There are findings suggesting that some people with developmental prosopagnosia also have other difficulties, or comparable strengths, in other domains like literacy, math, navigation etc. Therefore, we are interested in exploring this link and in this way adding further to the knowledge about developmental prosopagnosia.

About the project

In this research project, the aim is to investigate the subjective reports of cognitive difficulties and strengths by people with developmental prosopagnosia. This will be done through a custom-made questionnaire that contains questions regarding aspects of general health and questions about certain cognitive domains. A standard prosopagnosia questionnaire is also included. The full questionnaire takes **max. 15-20 minutes** to fill out.
There are no risks or physical discomforts associated with participating. All information provided in the questionnaire is anonymous, handled in accordance with Danish law about data protection and will only be used for research. Due to the anonymous nature of the data, it is not possible in any way to link any individual to a set of responses, and therefore it is not possible to withdraw your participation, once you have filled out the questionnaire. The project has received ethical approval

by University of Copenhagen, and is carried out with Professor Randi Starrfelt as principal investigator.

You can participate in the project, if you:

- Are older than 18 years of age
- Have been diagnosed with developmental prosopagnosia, either by a health professional or a researcher, OR suspect that you might suffer from developmental prosopagnosia due to everyday difficulties recognizing faces
- Have normal vision (either with or without the use of glasses/contact lenses)
- Do not currently have, and have not previously suffered from, brain injury or disease, i.e. stroke or severe head trauma or dementia

How can I participate?

If you wish to participate, you must read the above information and indicate that you have understood it on the next page. It is completely voluntary to participate, and all data will be anonymous. Please ensure that you are in an undisturbed environment, and preferably alone, for the 15-20 minutes it takes to answer the questionnaire.

Questions

If you have any questions, or if you are interested in reading the potential publication from this project, please contact Nanna Svart at (e-mail address provided originally) or professor Randi Starrfelt at (e-mail address provided originally).

The Difficulties and Abilities in Developmental Prosopagnosia Questionnaire

Item Number	Question	Answer Format	Questionnaire Section
-	I confirm that I have read and understood the information sheet for the project (previous page), and I am aware that I can ask questions by e-mail before participating.<br><br>If you have any questions, please contact Nanna Svart or professor Randi Starrfelt	Yes/No	Consent

-	I understand that the collected anonymous data can be used in publications, but cannot be linked to me personally. This means that I cannot withdraw my participation once I have filled out the questionnaire.	Yes/No	Consent
-	I understand that my participation in this project is voluntary, and I agree to take part in the study.	Yes/No	Consent
**1**	Have you been formally diagnosed with prosopagnosia (face blindness) by a healthcare professional, or tested by a researcher resulting in test scores indicating prosopagnosia?	Yes, please specify (free text) /No/	General information and health (GIH)
**2**	Do you have other diagnoses? Particularly related to vision, mental illness, learning difficulties, sensory disorders, developmental disorders, motor disorders, etc.	No / Dyslexia / ADHD/Autism spectrum disorder/ Dyscalculia/ Other, please specify (free-text)	GIH
**3**	Have you previously suffered from, or do you currently suffer from, any neurological illness, dementia or brain damage?	Yes, please specify_/No<br><br>(If a respondent answered yes, they would not be able to fill out the rest of the questionnaire, but could specify their condition)	GIH
**4**	How old are you?	18-24/ 25-34/ 35-44/ (...)/ 75 years or older	GIH
**5**	Is English your first language?	Yes/No	GIH
**6**	Do you have normal vision or corrected to normal vision with glasses or contact lenses?	Yes/No	GIH
**7**	Are you right- or left-handed?	Right / Left / Both (Ambidextrous)	GIH

**8**	My face recognition ability is worse than most people	Likert 1-5 (1: Strongly Disagree – 5: Strongly agree)	PI-20 – Item 1
**9**	I have always had a bad memory for faces	Likert 1-5	PI-20 - Item 2
**10**	I find it noticeably easier to recognize people who have distinctive facial features	Likert 1-5	PI-20 – Item 3
**11**	I often mistake people I have met before for strangers	Likert 1-5	PI-20 – Item 4
**12**	When I was at school I struggled to recognize my classmates	Likert 1-5	PI-20 - Item 5
**13**	When people change their hairstyle, or wear hats, I have problems recognizing them	Likert 1-5	PI-20 – Item 6
**14**	I sometimes have to warn new people I meet that I am ‘bad with faces’	Likert 1-5	PI-20 – Item 7
**15**	I find it easy to picture individual faces in my mind	Likert 1-5	PI-20 – Item 8 (reversed)
**16**	I am better than most people at putting a ‘name to a face’	Likert 1-5	PI-20 – Item 9 (reversed)
**17**	Without hearing people’s voices, I struggle to recognize them	Likert 1-5	PI-20 – Item 10
**18**	Anxiety about face recognition has led me to avoid certain social or professional situations	Likert 1-5	PI-20 – Item 11
**19**	I have to try harder than other people to memorize faces	Likert 1-5	PI-20 – Item 12
**20**	I am very confident in my ability to recognize myself in photographs	Likert 1-5	PI-20 – Item 13 (reversed)
**21**	I sometimes find movies hard to follow because of difficulties recognizing characters	Likert 1-5	PI-20 – Item 14

**22**	My friends and family think I have bad face recognition or bad face memory	Likert 1-5	PI-20 – Item 15
**23**	I feel like I frequently offend people by not recognizing who they are	Likert 1-5	PI-20 – Item 16
**24**	It is easy for me to recognize individuals in situations that require people to wear similar clothes (e.g. suits, uniforms, swimwear)	Likert 1-5	PI-20 – Item 17 (reversed)
**25**	At family gatherings, I sometimes confuse individual family members	Likert 1-5	PI-20 – Item 18
**26**	I find it easy to recognize celebrities in 'before-they-were-famous' photos, even if they have changed considerably	Likert 1-5	PI-20 – Item 19 (reversed)
**27**	It is hard to recognize familiar people when I meet them out of context (e.g. meeting a work colleague unexpectedly while shopping)	Likert 1-5	PI-20 – Item 20
**28**	I am good at understanding and following route descriptions	Likert 1-7 (1: Not at all applicable to me – 7: Fully applicable to me)	The Wayfinding Questionnaire (Original item 21)
**29**	I can always orient myself quickly and correctly when I am in an unknown environment	Likert 1-7	The Wayfinding Questionnaire (Original item 8)
**30**	I can easily find the shortest route to a known destination	Likert 1-7	The Wayfinding Questionnaire (Original item 26)
**31**	I can usually recall a new route after I have walked it once	Likert 1-7	The Wayfinding Questionnaire (Original item 18)

**32**	When I am in a building for the first time, I can easily point to the main entrance of this building	Likert 1-7	The Wayfinding Questionnaire (Original item 2)
**33**	How much extra help did you need when learning to read in elementary school?	Likert 1-5 (Help from: 1. No help, 2. Friends, 3. Teachers/parents, 4. Tutors or special class *1 year*, 5. Tutors or special class *2 or more years*)	The Adult Reading History Questionnaire (Original item 3)
**34**	How would you compare your reading skill to that of others in your elementary classes?	Likert 1-5 (1: Above average, 3: Average, 5: Below average)	The Adult Reading History Questionnaire (Original item 6)
**35**	How much difficulty did you have learning to spell in elementary school?	Likert 1-5 (1: None, 3: Some, 5: A great deal)	The Adult Reading History Questionnaire (Original item 13)
**36**	How much difficulty did you have learning to read in elementary school?	Likert 1-5 (1: None, 3: Some, 5: A great deal)	The Adult Reading History Questionnaire (Original item 2)
**37**	How would you compare your current reading speed to that of others of the same age and education?	Likert 1-5 (1: Above average, 3: Average 5: Below average)	The Adult Reading History Questionnaire (Original item 11)
**38**	As a child, did you make careless errors in math, such as adding when the sign indicated subtraction?	Likert 1-5 (1: Strongly Disagree – 5: Strongly agree)	Adapted from The Colorado Learning

			Difficulty Questionnaire (Original item 19)
**39**	As a child, did you have trouble learning new math concepts?	Likert 1-5 (1: Strongly Disagree – 5: Strongly agree)	Adapted from The Colorado Learning Difficulty Questionnaire (Original item 20)
**40**	Do you have difficulties estimating quantities, e.g. how many coins are lying on a table?	Likert 1-5 (1: Strongly Disagree – 5: Strongly agree)	Estimation of Numerosities Item
**41**	Are there things that you are particularly good at, where you experience performing better than others?<br><br>*Please consider and elaborate on any special skills or talents you might have, for example within the areas of mathematical abilities, memory, navigation, visualizing images or objects, literacy, sports or musical talents.*	Free text	
**42**	Are there things or situations, where you experience performing worse than others?<br><br>*Please consider and elaborate on any difficulties you might have, for example within the areas of math, memory, navigation, visualizing images or objects, literacy, sports or tone deafness.*	Free text	
**43**	Do you have any of the following conditions related to cognitive strengths or difficulties?	1. No<br><br>2. Aphantasia (inability to visualize things in detail in your mind)	

		3. Synesthesia (merging of sensations, e.g. seeing numbers/letters in colours or experiencing music as physical sensations)<br><br>3. Object agnosia (problems recognising specific types of objects, e.g. cars)<br><br>4. Memory problems (remembering things, details, or personal past events)<br><br>5. Other (free text)	

Last Page of the Full Questionnaire

Thank you for taking your time to fill out this questionnaire as it is valuable information to us. We appreciate your contribution to research in developmental prosopagnosia!

If you have any questions please don't hesitate Nanna Svart at (e-mail address provided originally) or professor Randi Starrfelt at (e-mail address provided originally). Should the questions have initiated any emotional discomfort you are also more than welcome to contact us. It is not unusual, should you feel a little uneasy, but it is important to stress that this is not a diagnostic questionnaire, but a questionnaire about your subjective self-evaluation.

Last Page of Questionnaire if Respondent Answers 'Yes' to item 3 (brain damage)

Thank you! We appreciate that you took your time to fill out this questionnaire as it is valuable information to us. In this research project, we are interested in looking into people with

developmental prosopagnosia without any known history of brain injury or brain disease. We are aware that many people with difficulties recognising faces have never been formally diagnosed, and that some are unsure whether they have had, e.g. an injury in childhood, so that some of these questions can be hard to answer.

If you have any questions please don't hesitate Nanna Svart at (e-mail address provided originally) or professor Randi Starrfelt at (e-mail address provided originally). Should the questions have initiated any emotional discomfort you are also more than welcome to contact us. It is not unusual, should you feel a little uneasy, but it is important to stress that this is not a diagnostic questionnaire, but a questionnaire about your subjective self-evaluation.

Last Page of Questionnaire if Respondent Answers 'No' to any Consent Items
You have answered no to some of our consent question regarding the collection of anonymous data for research, and hence cannot proceed with the questionnaire.
If you have any questions, please contact Nanna Svart at (e-mail address provided originally) or professor Randi Starrfelt at (e-mail address provided originally).

Specific References

<u>The 20-item Prosopagnosia Index:</u>

Shah, P., Gaule, A., Sowden, S., Bird, G., & Cook, R. (2015). The 20-item prosopagnosia index (PI20): a self-report instrument for identifying developmental prosopagnosia. *Royal Society open science, 2*(6), 140343. doi:10.1098/rsos.140343

<u>The Wayfinding Questionnaire:</u>

Claessen, M. H. G., Visser-Meily, J. M. A., de Rooij, N. K., Postma, A., & van der Ham, I. J. M. (2016). The Wayfinding Questionnaire as a Self-report Screening Instrument for Navigation-related Complaints After Stroke: Internal Validity in Healthy Respondents and Chronic Mild Stroke Patients. *Archives of Clinical Neuropsychology, 31*(8), 839-854. doi:10.1093/arclin/acw044

<u>The Adult Reading History Questionnaire:</u>

Lefly, D. L., & Pennington, B. F. (2000). Reliability and Validity of the Adult Reading History Questionnaire. *Journal of Learning Disabilities, 33*(3), 286-296. doi:10.1177/002221940003300306

The Colorado Learning Difficulty Questionnaire:

Willcutt, E. G., Boada, R., Riddle, M. W., Chhabildas, N., DeFries, J. C., & Pennington, B. F. (2011). Colorado Learning Difficulties Questionnaire: validation of a parent-report screening measure. *Psychological Assessment, 23*(3), 778-791. doi:10.1037/a0023290
